# Timing matters: a prospective investigation of father absence and pubertal timing in girls and boys

**DOI:** 10.1186/s13293-026-00868-y

**Published:** 2026-03-19

**Authors:** Taylor M. Drazan, Natasha Chaku

**Affiliations:** Indiana University Bloomington, Bloomington, 47405 IN USA

## Abstract

**Background:**

Puberty is beginning earlier across cultures and geographic regions. Father absence is one of the most robust psychosocial predictors of earlier pubertal timing. Yet the importance of its timing (i.e., when absence and presence are most impactful), variability (i.e., fluctuations in absence and presence), and consistency (i.e., the persistence of absence or presence) remains understudied, as do the developmental mechanisms that underlie associations between father absence and pubertal timing.

**Methods:**

Data were obtained from the Future of Families and Child Wellbeing Study, a prospective cohort of 4,898 participants. Father absence was indexed via mother-reports at birth, and ages 1, 3, 5, and 9. Pubertal timing was indexed via breast development in girls, facial hair growth in boys, and pubic and underarm hair in both at age 9. Relative maturation was also assessed at ages 9 and 15. Child body mass index, father depression, and material hardship were examined as possible mediators at age 5.

**Results:**

Four distinct patterns of father absence emerged, representing fathers who were (1) consistently absent, (2) absent early in life but present later in childhood, (3) present early in life but absent later in childhood, and (4) consistently present. Consistent father absence *and father absence in the first year of life only* were associated with earlier breast development in girls and earlier facial hair growth in boys, but not earlier pubic and underarm hair growth; father absence was inconsistently associated with indicators of relative maturation. None of the proposed mediators significantly mediated these associations.

**Conclusions:**

The findings from this prospective cohort study suggest that father absence, particularly in the first year of life, is associated with signs of earlier pubertal timing in both girls and boys. These patterns are consistent with sensitive period hypotheses, but must be further investigated in the context of broader signals of adversity and resilience.

**Supplementary Information:**

The online version contains supplementary material available at 10.1186/s13293-026-00868-y.

## Abstract (plain language summary)

Across countries, children are starting puberty at younger ages than in the past. Entering puberty earlier than same-aged, same-sexed peers is linked to greater risk for depression, risk taking, teenage pregnancy, and even long-term health problems such as heart disease. Although biological factors, such as body weight and genetics, play a role in when puberty begins, these factors do not fully explain why some people start puberty earlier than others. Social and family experiences also appear to matter. One important factor may be whether a father is present in the household during childhood. Using data from nearly 5,000 families followed from birth to age 15, we identified four patterns of father absence. Children whose fathers were consistently absent, or absent during the first year of life only, were more likely to show signs of pubertal development at age 9 than those whose fathers were present in the household throughout childhood. These patterns were similar for both girls and boys, but they varied across pubertal milestones. Our findings suggest that the first year of life may be a particularly important window for development. Supporting stable, healthy family environments early in life may be beneficial for children’s later development.

**Supplementary Information:**

The online version contains supplementary material available at 10.1186/s13293-026-00868-y.

## Highlights

- Puberty is beginning at younger ages across cultures and geographic regions.
- Father absence is a robust psychosocial predictor of earlier pubertal timing in girls, with mixed evidence in boys.
- Repeated measures latent class analyses identified four trajectories of father presence: (1) consistently absent, (2) absent early in life but present later in childhood, (3) present early in life but absent later in life, and (4) consistently present.
- Girls and boys with consistently absent fathers or fathers who were absent early in childhood demonstrated earlier signs of pubertal timing.

**Supplementary Information:**

The online version contains supplementary material available at 10.1186/s13293-026-00868-y.

## Introduction

Puberty is a complex biopsychosocial process that marks the transition from childhood to adulthood. The timing and progression of puberty profoundly shapes developmental pathways and has significant implications for health outcomes [1, 2]. For example, earlier pubertal timing (i.e., relative maturation compared to same-aged, same-sexed peers) tends to culminate in a variety of negative outcomes in adulthood. Specifically, women who develop earlier are at increased risk for depression and obesity in young adulthood, two key markers of future disability;[3, 4] they are also at increased risk for cardiometabolic disorders (including Type 2 diabetes or cardiovascular disease) and even reproductive and breast cancers in midlife [5, 6]. Less work has considered men’s pubertal timing, but recent work suggests that men who develop earlier are more likely to be overweight and abuse substances in young adulthood which could have lifelong effects on future health and wellbeing [7, 8].

Girls typically begin puberty between 8–13-years-old and boys, two to three years later. Although 50–80% of individual variation in pubertal timing is heritable, average pubertal timing has advanced by three months per decade in girls and 1.5 months per decade in boys [9–11] While most of these changes have been attributed to increases in adiposity and changes in nutrition, psychosocial factors and family contexts also influence pubertal timing [12, 13]. For instance, Life History Theory suggests that early life stressors, and especially unstable family environments (e.g., marital disharmony, single parent households), may regulate subsequent life history strategies, cueing earlier pubertal timing [14, 15]. The putative mechanisms linking these early life stressors and earlier pubertal timing include harsher environmental conditions (e.g., lower socioeconomic status, lower caregiving quality) that prompt adaptation to the prospect of later adversity, [13] stress-related alterations in hypothalamic-pituitary-adrenal (HPA) axis functioning that are associated with downstream reproductive maturation, [16] and increases in adiposity which disproportionally contribute to earlier puberty in girls compared to boys [17].

Of known early life stressors, father absence in the household represents an important feature of the proximal childhood environment, [17] and recent evidence suggests it is among the most robust psychosocial predictors of earlier pubertal timing in girls [18, 19]. Indeed, girls with absent fathers are twice as likely to experience menarche prior to 12 years old. Although the observed effects are generally small (*d*s = 0.10–0.20), associations between father absence and earlier pubertal timing are generally consistent across prospective and retrospective studies and across North American and non-North American contexts [17, 20, 21]. At the same time, however, the literature reveals substantial heterogeneity, including a considerable number of nonsignificant findings and some opposing effects [22–24]. Further, effects often differ among girls and boys (when boys are even assessed) and typically suggest that associations between father absence and pubertal timing are weaker and less consistent among boys than girls [24–26].

Despite the importance of father absence for understanding pubertal development, prior research has been limited in several ways. First, past research has primarily focused on cross-sectional studies that cannot adequately assess the importance of timing (i.e., when a father’s absence or presence is most impactful), stability (i.e., fluctuations in a father’s absence and presence), or consistency (i.e., persistence of father’s absence or presence). Indeed, key theories in developmental science suggest that there are sensitive windows when positive and negative experiences may have an outsized impact on developing physiological and neurobiological systems [27, 28] For example, absence during the first year of life, when the most dramatic and rapid brain development occurs, may disrupt the development of key cognitive control structures related to later health behaviors, [29] but absence in middle childhood during the reactivation of HPA axis, may disrupt key stress systems related to health as well [30]. Thus, the impact of father absence may depend on developmental stage, but *most studies rely on single time-point designs*, which fail to capture how father absence, and its impact, may change over infancy and childhood.

Second, most research has not used concurrent indicators of pubertal timing, instead relying on retrospective reporting of menarche, which is socially salient and well-remembered, [31] but occurs relatively late in puberty and has no comparable indicator in boys [32]. Thus, not only is menarche a poor indicator of earlier pubertal milestones, but the focus on menarche has led to a comparative lack of knowledge on father absence and boy’s pubertal development [12, 18]. This is particularly problematic because key pubertal events for boys are often less visible and more gradual than discrete events like menarche [33]. These concerns could be overcome by using earlier pubertal milestones in girls such as thelarche (or breast development),[34] socially salient ones in boys such as facial hair growth, [33] or comparable indicators in both girls and boys such as pubic and underarm hair growth [12]. Additionally, subjective measures of pubertal timing such as social comparison questions that ask youth (or parents) to compare their development to same-aged, same-sexed peers are excellent alternatives that capture both physical development and their social context [35].

Finally, by focusing on form (i.e., family structure) rather than process (i.e., how families organize themselves), prior research has offered little insight into the developmental pathways through which family contexts influence pubertal transitions. Both familial structures and pubertal development are dynamic, ongoing processes shaped by culminative experiences that unfold over time [36]. Yet, previous research has rarely examined the temporal ordering, or psychosocial mechanisms, that underlie associations between father absence and pubertal timing. For instance, socioeconomic stress may accelerate biological maturation by increasing exposure to adversity and reducing parental availability;[37] father characteristics such as their mental health, warmth, or parenting involvement may influence both family stability and stress regulation;[38] and youth health and nutritional factors may interact with these contextual features to shape the timing and pace of pubertal development [17]. Exploring these potential mechanisms could offer insight into when and how family dynamics become biologically embedded, identify sensitive windows for prevention, and clarify the contextual factors that shape individual variation in pubertal timing.

To begin to address these limitations, the current study used a large United States cohort with repeated measures of father absence and multiple indicators of pubertal timing to examine how timing, stability, and consistency of father absence was associated with individual variation in pubertal timing across girls and boys. Material hardship, father mental health, and child Body Mass Index (BMI) were considered as mediators. We hypothesized that youth who experienced consistent father absence over childhood would be more likely to report earlier pubertal timing than those with consistently present fathers. We additionally hypothesized that material hardship would mediate associations between father absence and pubertal timing.

## Methods

### Data

Data for this analysis were drawn from the Future of Families & Child Wellbeing Study, [39] a large, longitudinal study that tracked 4,898 children from 20 cities across the United States. The study aimed to understand how environmental and social factors influenced the development of at-risk children, with a particular focus on the relationship between unmarried parents and child outcomes. Data collection began at the focal child’s birth between 1998 and 2000, when parents or caregivers completed questionnaires. Follow-up assessments were conducted at ages 1, 3, 5, 9, and 15, with additional questionnaires completed by the focal child at ages 9 and 15. The original data collection was approved by the Princeton University Institutional Review Board, and this study was approved by the Institutional Review Board at Indiana University. Further details about this study design can be found at https://ffcws.princeton.edu.

### Participants

The sample included 4,898 children (2,341 girls); 47.49% of the sample reported non-Hispanic Black race/ethnicity, 27.28% reported non-White Hispanic race/ethnicity, 21.03% reported non-Hispanic White race/ethnicity and 3.9% reported another race/ethnicity. At birth, 34.69% of mothers reported having less than a high school education, 30.22% a high school education, 24.28% some college education, and 10.7% a college degree or higher. Detailed information about sample characteristics is presented in Table 1.

**Table 1:** Full sample characteristics and descriptive information (*N* = 4,898)

Birth	Age assessed	*n*	%
Sex	birth		
Female		2341	47.8%
Male		2557	52.2%
Mother’s education	birth		
Did not complete high school		1699	34.69%
Completed high school/GED		1480	30.22%
Some college		1189	24.28%
Bachelors/post-graduate degree		524	10.70%
* Missing*		6	0.11%
Race/Ethnicity	birth		
Non-Hispanic Black		2326	47.49%
Non-White Hispanic		1336	27.28%
Non-Hispanic White		1030	21.03%
Another race/ethnicity		194	3.9%
* Missing*		12	0.3%
Marriage status	birth		
Married		1187	24.23%
Unmarried		3710	75.75%
* Missing*		1	0.02%
Raised by non-biological parents	age 9		
Child raised by biological parents		2579	52.65%
Child raised by non-biological parents		69	1.41%
* Missing*		2250	45.94%
Mother presence	age 9		
Mother present		3272	66.8%
Mother absent		241	4.83%
* Missing*		1385	28.37%
Cohabiting with partner	age 9		
Non-biological partner present in household		428	8.74%
Non-biological parent not present in household		3087	63.03%
* Missing*		1383	28.23%
Pubertal status	age 9		
Begun breast development		927	18.92%
Begun facial hair growth		112	2.29%
Social comparison of pubertal status	age 9		
Earlier than peers		616	12.57%
Similar to peers		2550	52.06%
Later than peers		448	9.14%
* Missing*		1284	26.23%
Social comparison of pubertal status	age 15		
Earlier than peers		773	15.78%
Similar to peers		1978	40.38%
Later than peers		632	12.9%
* Missing*		1515	30.94
Child BMI	age 5		
Normal weight		1411	28.81%
Overweight		752	15.35%
* Missing*		2735	55.84%
Father depression	age 5		
Meets depression criteria		364	7.43%
Does not meet depression criteria		2782	56.80%
* Missing*		1752	35.77%
		*M*	*SD*
Maternal age	birth	21.57	5.24
Material Hardship	age 5	2.91	2.77

Missing: number of individuals who did not complete the assessment. N: sample size. %: percentage. M: mean. SD: standard deviation

### Measures

#### Pubertal timing

Pubertal timing was assessed at ages 9 and 15 via parent- and child-report respectively. At age 9, the Pubertal Developmental Scale (PDS) [40] was administered to parents. The PDS is a well validated, widely used self-report measurement of physical development that correlates with biological markers of pubertal development [41]. Sex-specific items “Would you say that her breasts have started to grow?” (thelarche, girls only) and “Has he started to grow hair on his face?” (facial hair growth, boys only) as well as a sex-neutral item “Would you say that growth of his/her underarm or pubic hair has started yet?” (pubic and underarm hair) were used to indicate whether pubertal development had begun by age 9 (0 = *Development has not yet begun*, 1 = *Development has begun*). Additionally, at age 9, parents evaluated whether they perceived their child’s physical development as earlier, about the same, or later compared to same-aged, same-sexed peers. At age 15, the focal child answered a similar question about their own development. Responses indicating “somewhat earlier” or “much earlier” were categorized as earlier pubertal timing, whereas responses of “about the same as other boys/girls” were coded as on-time development, and “somewhat later” or “much later” were categorized as later development.

Multiple, single PDS items were selected to capture distinct aspects of early development, while avoiding the complications of the total PDS score, which averages across many pubertal milestones, all of which normatively occur at different points during puberty [42]. Thelarche and facial hair growth were selected because they index the most visible features of puberty for girls and boys respectively and often carry social significance for youth and their parents [32]. These indicators reflect gonadal processes, but occur at different points of development across sexes: Specifically, thelarche is one of the first indicators of pubertal development whereas facial hair growth occurs much later in puberty [18]. Pubic and underarm hair growth was thereby included as an additional indicator of pubertal development because pubic and underarm hair tend to emerge at a comparable point of development for girls and boys. However, the development of pubic/underarm hair reflects a different physiological process (namely, adrenarche) and is not as socially salient or well-observed by parents [41, 43]. Finally, peer comparisons in pubertal development were included because these relative measures index some of the social aspects of pubertal development well and are common alternatives when longitudinal puberty data is unavailable [10].

#### Father absence

Father absence was characterized by mother reports on their relationship with the father and their presence in the household at birth and ages 1, 3, 5, and 9. Categories included “married”, “romantic cohabiting”, “no relationship”, “friends”, “separated/widowed/divorced”, “romantic no visit”, and “dad unknown”. Following previous research, [44] categories were collapsed into a single binary indicator of *presence in the household* (i.e., “married” and/or “romantic cohabiting”) or *absence in the household* (i.e., “no relationship,” “friends,” “separated/widowed/divorced,” “romantic no visit,” “romantic some visit,” and “dad unknown”).

#### Psychosocial characteristics

 Mediating variables were assessed at age 5 and included material hardship, father depression, and child BMI. Material hardship comprised thirteen items (i.e., “In the past year, did your children go hungry?”) which were summed to create a composite score where higher scores indicated more reported hardship [45]. Material hardship was reported on by the father when available, and mothers if the father-report was missing at age 5. Father depression comprised twelve depression items (e.g., “Did you feel more tired and low on energy?”) which were averaged to create a composite score; higher scores indicated more self-reported depression [46]. Child’s BMI was reported by the primary caregivers (mostly mothers). BMI was categorized as normal or overweight based on age- and sex-specific growth charts from the CDC; overweight was defined as a BMI at or above the 85th percentile for boys and girls [47].

#### Covariates

 Several covariates were included, such as whether the child was raised by non-biological parents (i.e., “Who does the child usually live with?”), whether the mother was present in the household the child lived in (“How much of the time does the child live with you?”), and whether the mother was cohabiting with a partner other than the father. Additionally, we included maternal age at birth and race/ethnicity, categorized as Black Non-Hispanic, Hispanic, White Non-Hispanic, or another race/ethnicity.

### Analytic plan

A repeated measures latent class analysis (RMLCA) in Mplus Version 8.0 [48] was used to identify patterns of father absence over time and examine their associations with pubertal timing in the full sample of girls and boys. RMLCA is a person-centered approach that groups individuals into latent subgroups based on similarities in their observed responses across time [49]. This approach allowed us to identify qualitatively different patterns of father presence or absence across development without aggregating or assuming homogeneity. We iteratively tested solutions starting from a one-class model and adding additional classes until model fit did not improve, as indicated by the Sample-Adjusted Bayesian Information Criteria (sa*BIC*) [50]. The optimal model was chosen based on the minimum sa*BIC* value and the inclusion of at least 5% of the sample in each class[51].

To explore associations between latent class membership and pubertal timing (thelarche for girls, facial hair for boys, and pubic and underarm hair as well as social comparison for both), the three-step Bolck, Croon, and Hagenaars (BCH) procedure [52] was employed separately by sex. Specifically, after the most likely latent class membership was estimated, mean differences in each indicator of pubertal timing were assessed by class membership while adjusting for classification uncertainty and all covariates. The statistical significance of class-specific mean differences were assessed using Wald tests of mean equality,[52] and effect sizes for differences in distal outcomes were calculated using Cohen’s *d*, with values of 0.2, 0.5, and 0.8 representing small, medium, and large effect sizes, respectively [53]. Full Information Maximum Likelihood (FIML) estimation was used to address missing data.

In addition to examining distal outcomes, mediation analyses were conducted to investigate whether material hardship, father mental health, or child BMI explained the association between father absence class membership and pubertal timing. In these analyses, the mediator was modeled as an outcome regressed on different indicators of pubertal timing within each latent class. Indirect effects were specified using the MODEL CONSTRAINT command in Mplus, which was used to define new parameters representing the product of differences in the intercepts and slopes across classes [54]. Statistical significance for these indirect effects were assessed via two-tailed *t*-tests, and 95% confidence intervals were computed to inform the interpretation of the effect.

## Results

**Fig. 1:**
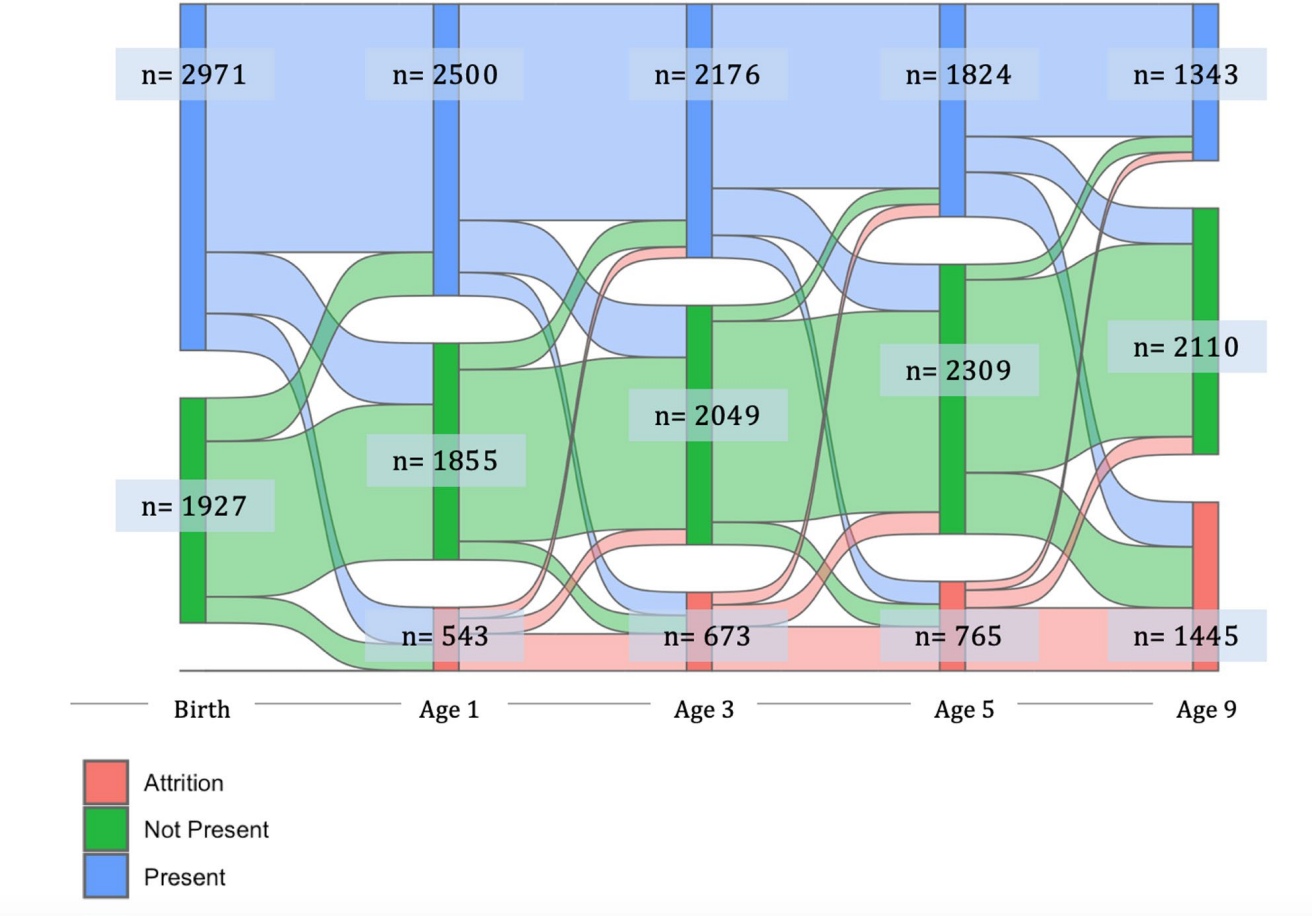
Sankey plot depicting transitions in father presence, absence, and attrition from birth through Year 9. Blue: Father present in the household, Green: Father not present in the household. Red: Family was not in the study that year. The width of each bar reflects the number of participants transitioning between presence and absence. *n*: 4,898

Figure 1 illustrates significant changes in father presence and absence over time. Father absence increased from birth to age 9, and most absent fathers left the household between birth and age 1. However, fathers continued to leave and/or return to the household in early- and mid-childhood demonstrating the variability of presence and absence across development.

### Patterns of father absence across time

The fit statistics for the repeated measures latent class models are presented in Supplemental Table 1. The best-fitting model revealed four distinct classes representing fathers who were (1) consistently absent (39.3%), (2) absent early but present later (6.2%), (3) present early but absent later (15.6%), and (4) consistently present (38.9%). The consistently absent class represented fathers who did not live in the household at any time from birth to 9-years-old. The early absence/later presence represented fathers who were absent at birth and 1-years-old, with substantial increases in presence during later years. Conversely, the early presence/later absence class represented fathers who were predominantly present at birth and 1-years-old, followed by a notable decline in their presence during later years. The consistently present class represented fathers who were present across all time points. The percent of fathers present in each class across time is presented in Supplemental Table 2.

### Father absence and pubertal timing in girls and boys

**Fig. 2:**
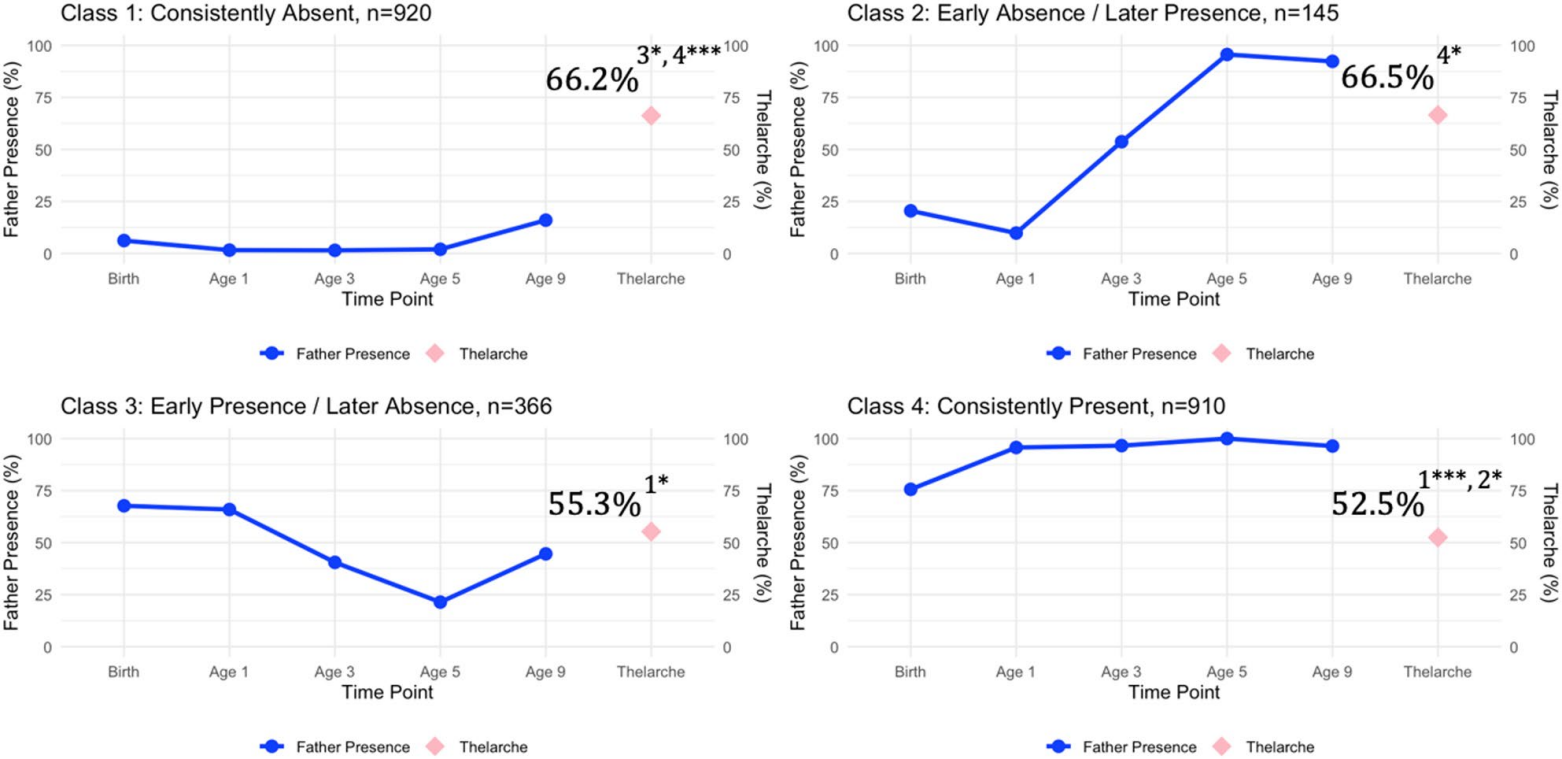
Father Presence and Thelarche at Age 9. The relationship between father absence across five time points (birth, ages 1, 3, 5, and 9) and thelarche at age 9 for each latent class. The solid blue line indicates the percent of father’s present across time points; the pink diamond marker represents the estimated percentage of individuals within each latent class who experienced thelarche at age 9. (**A**) 66.2% of girls in the consistent absent class reported thelarche at year 9. (**B**) 66.5% of girls in the early absence/later presence class reported thelarche at year 9. (**C**) 55.3% of girls in the early presence/later absence class reported thelarche at year 9. (**D**) 52.5% of girls in the consistently present class reported thelarche at year 9. Subscripts represent significant differences between latent classes. ^1^ = significantly different from the consistently absent class. ^2^ = significantly different from the early absence/later presence class. ^3^ = significantly different from the early presence/later absence class. ^4^ = significantly different from the consistently present class. * *p* <.05, ** *p* <.01, *** *p* <.001

Associations between father absence and pubertal timing were investigated for each indicator of pubertal timing, starting with thelarche and facial hair growth. After adjusting for covariates, 66.2% of girls in the consistently absent class reported thelarche at age 9, compared to 66.5% in the early absence/later presence class, 55.3% in the early presence/later absence class, and 52.5% in the consistently present class (see Fig. 2). Girls with consistently absent fathers were significantly more likely to report thelarche at age 9 than girls whose fathers were present early in development (class 1 vs. class 3: *p* =.035, Cohen’s *d* = 0.11) or girls with consistently present fathers (class 1 vs. class 4: *p* =.001, Cohen’s *d* = 0.14). Girls whose fathers *returned to the household* were also significantly more likely to report thelarche at age 9 than girls with consistently present fathers (class 2 vs. class 4: *p* =.050, Cohen’s *d* = 0.14). There were no other significant differences in thelarche between classes (all *p*s > 0.05, Cohen’s *d*s = 0.001–0.11).

**Fig. 3:**
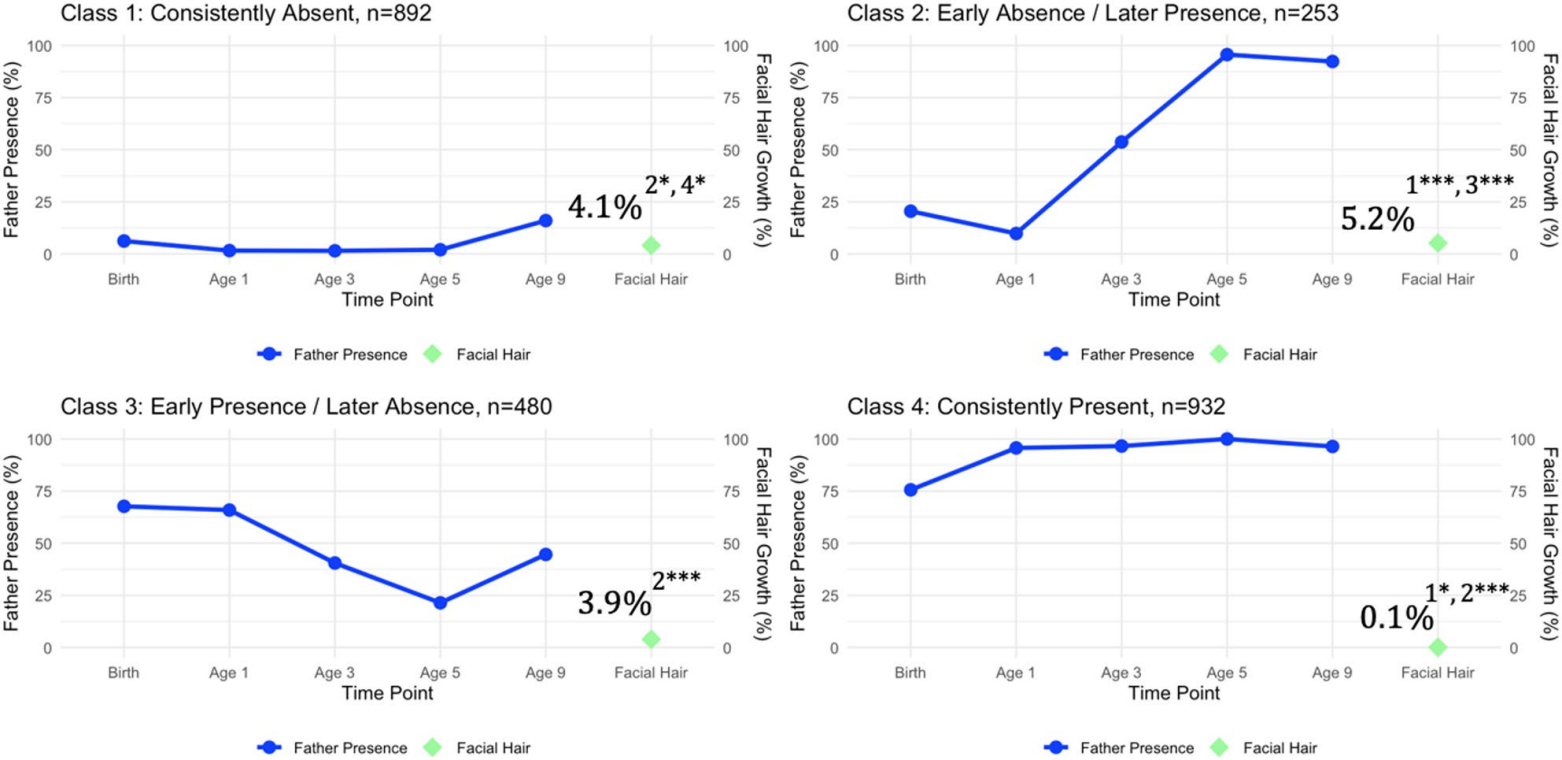
Father Presence and Facial Hair Growth at Age 9. The relationship between father presence across five time points (birth, ages 1, 3, 5, and 9) and facial hair growth at age 9 for each latent class. The solid blue line indicates the percent of father’s presence across time points; the green diamond marker represents the estimated percentage of individuals within each latent class who experienced facial hair growth at age 9. (**A**) 4.1% of boys in the consistent absent class reported facial hair growth at year 9. (**B**) 5.2% of boys in the early absence/later presence class reported facial hair growth at year 9. (**C**) 3.9% of boys in the early presence/later absence class reported facial hair growth at year 9. (**D**) 0.1% of boys in the consistently present class reported facial hair growth at year 9. Subscripts represent significant differences between latent classes. ^1^ = significantly different from the consistently absent class. ^2^ = significantly different from the early absence/later presence class. ^3^ = significantly different from the early presence/later absence class. ^4^ = significantly different from the consistently present class. * *p* <.05, ** *p* <.01, *** *p* <.001

After adjusting for covariates, 4.1% of boys in the consistently absent class reported facial hair growth at age 9, compared to 5.2% in the early absence/later presence class, 3.9% in the early presence/later absence class, and 0.1% in the consistently present class (see Fig. 3). Boys whose fathers were absent early in development were significantly more likely to report facial hair growth at age 9 than boys whose fathers were present early in development (class 2 vs. class 3: *p* <.001, Cohen’s *d* = 0.091), boys with consistently present fathers (class 2 vs. class 4: *p* <.001, Cohen’s *d* = 0.053), or boys whose fathers were consistently absent (class 1 vs. class 2: p = < 0.001, Cohen’s *d* = 0.093). Boys with consistently absent fathers were also significantly more likely to report facial hair growth at age 9 than boys with consistently present fathers (class 1 vs. class 4: *p* =.027, Cohen’s *d* = 0.041). There were no other significant differences in facial hair growth between classes (all *p*s > 0.05, Cohen’s *d*s = 0.002–0.038).

Then, associations between father absence and pubertal timing were investigated using an indicator of pubic and underarm hair growth. For girls, 72.7% in the consistently absent class reported pubic and underarm hair growth at age 9, compared to 88.8% in the early absence/later presence class, 74.6% in the early presence/later absence class, and 67.8% in the consistently present class. However, after adjusting for covariates, there were no significant differences in pubic and underarm hair growth by class (all *p*s > 0.05, Cohen’s *d*s = 0.001–0.039). For boys, 17.7% in the consistently absent class reported pubic and underarm hair growth at age 9, compared to 16.3% in the early absence/later presence class, 12.5% in the early presence/later absence class, and 19.4% in the consistently present class (see Fig. 5). Again, after adjusting for covariates, there were no significant differences in pubic and underarm hair growth by class (all *p*s > 0.05, Cohen’s *d*s = 0.008–0.041). See Supplemental Figs. 1–2 for additional details.

**Fig. 4:**
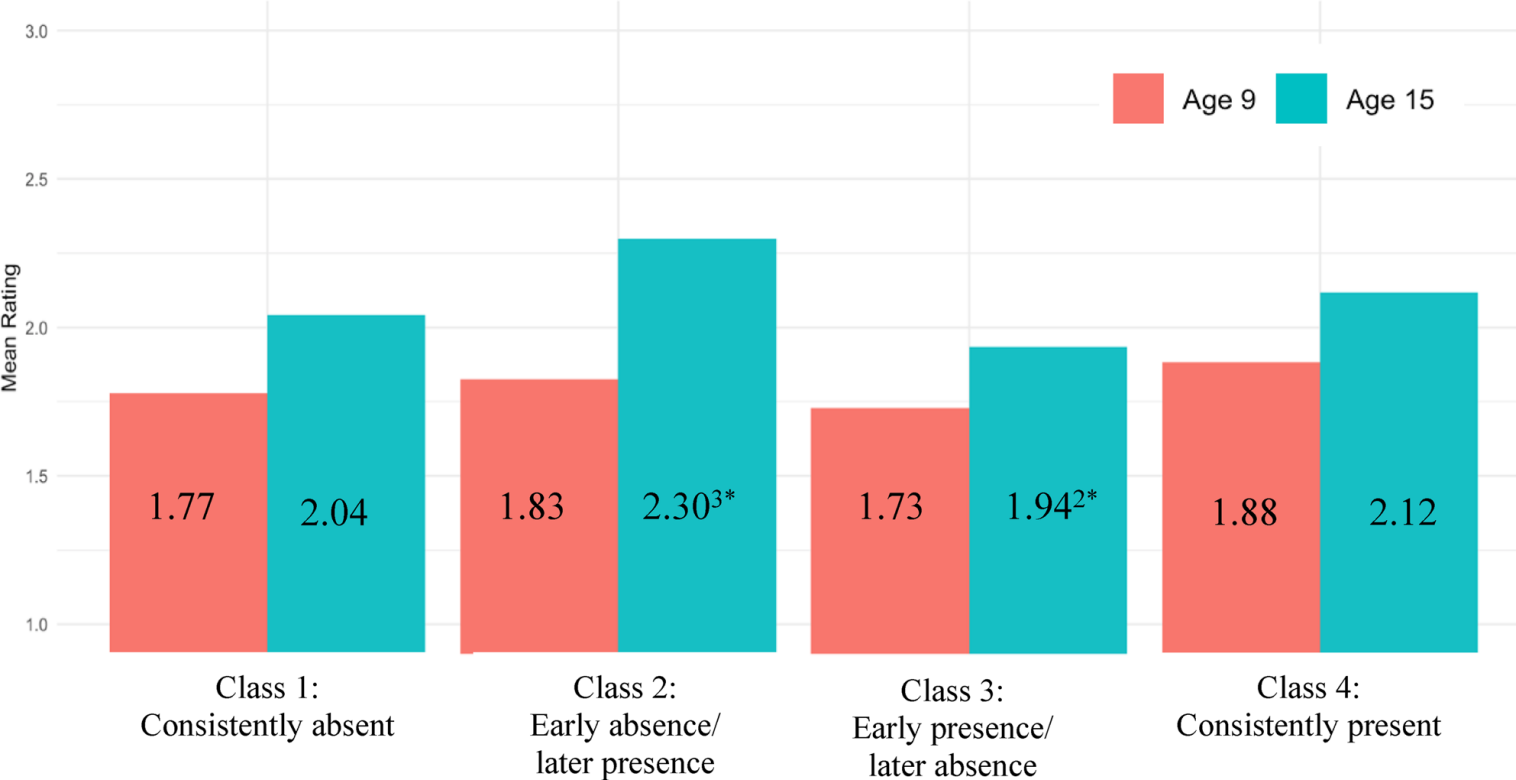
Girls’ pubertal timing by latent class and age. Ratings of pubertal development were: 1 = *earlier development compared to same-aged*,* same-sexed peers*, 2 = *similar development compared to same-aged*,* same-sexed peers*, or 3 = *later development compared to same-aged*,* same-sexed peers*. The red bars represent peer comparisons at year 9 reported by the primary caregiver. The blue bars represent peer comparisons at year 15 reported by the focal child. Each grouped bar depicts a different father absence class: (1) Consistently absent, (2) Early absence/Later presence, (3) Early presence/Later absence, and (4) Consistently present. Subscripts represent significant differences between latent classes. ^1^ = significantly different from the consistently absent class. ^2^ = significantly different from the early absence/later presence class. ^3^ = significantly different from the early presence/later absence class. ^4^ = significantly different from the consistently present class. * *p* <.05, ** *p* <.01, *** *p* <.001

Finally, associations between father absence and pubertal timing were investigated using social comparisons of pubertal development (reported by parents at age 9 and children at age 15). For girls, at age 9, there were no significant differences in parent-reported social comparisons across classes (all *p*s > 0.05, Cohen’s *d*s = 0.047–0.153). At age 15 though, there were significant differences in child-reported social comparisons across classes (see Fig. 4). Specifically, girls whose fathers were absent later in development (*M*_*timing*_ = 1.935, *SD* = 0.586) were more likely to report earlier timing compared to same-aged, same-sexed peers than girls whose fathers were absent earlier and then returned to the household (*M*_*timing*_ = 2.298, *SD* = 0.586; class 2 vs. class 3: *p* =.028, Cohen’s *d* = 0.363). There was also evidence that girls whose fathers were consistently absent were more likely to report earlier timing compared to same-aged, same-sexed peers than girls whose fathers were absent earlier and then returned to the household (class 1 vs. class 2: *p* =.050, Cohen’s *d* = 0.256).

**Fig. 5:**
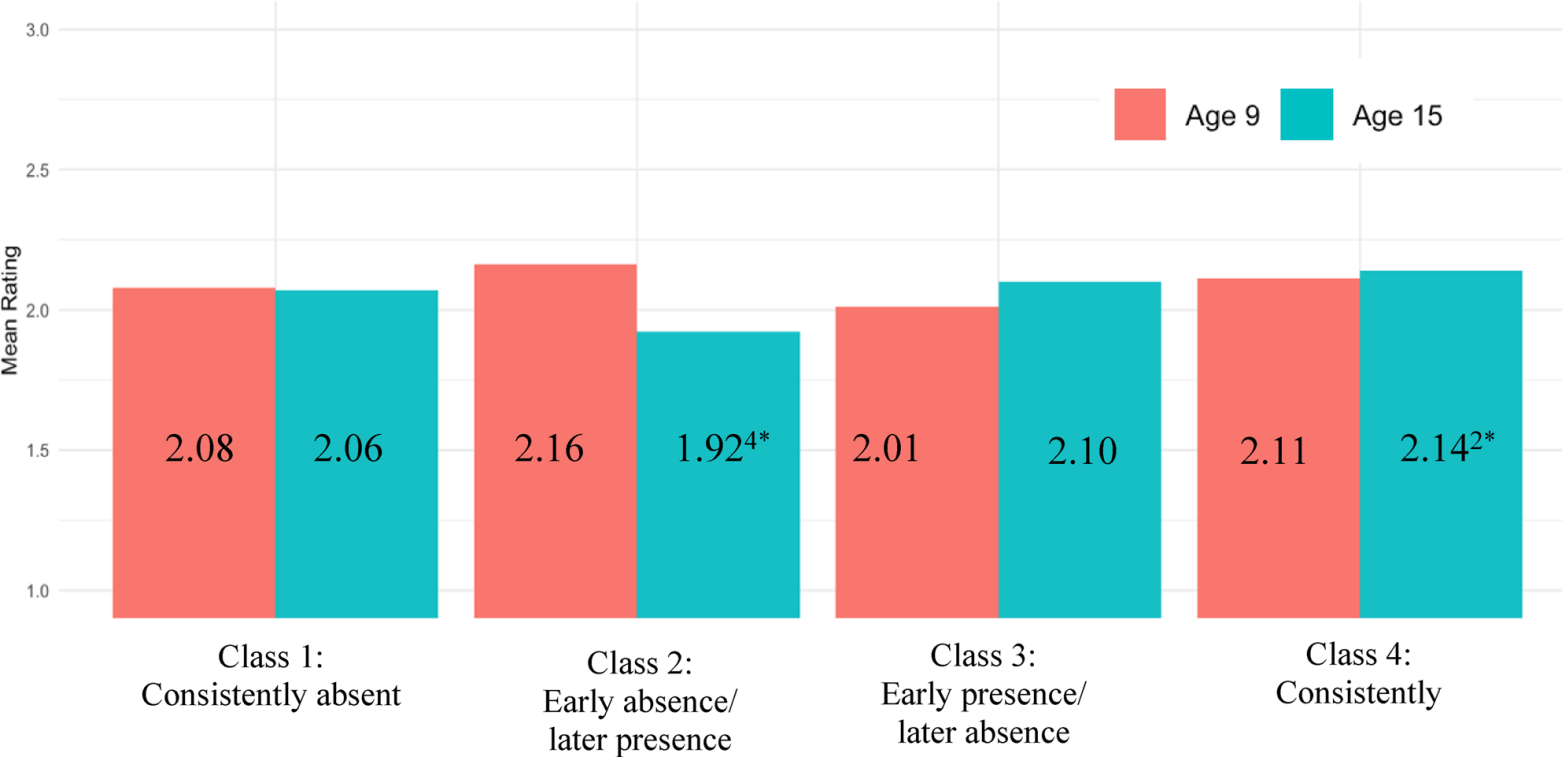
Boys’ pubertal timing by latent class and age. Ratings of pubertal development were: 1 = *earlier development compared to same-aged*,* same-sexed peers*, 2 = *similar development compared to same-aged*,* same-sexed peers*, or 3 = *later development compared to same-aged*,* same-sexed peers*. The red bars represent peer comparisons at year 9 reported by the primary caregiver. The blue bars represent peer comparisons at year 15 reported by the focal child. Each grouped bar depicts a different father absence class: (1) Consistently absent, (2) Early absence/Later presence, (3) Early presence/Later absence, and (4) Consistently present. Subscripts represent significant differences between latent classes. ^1^ = significantly different from the consistently absent class. ^2^ = significantly different from the early absence/later presence class. ^3^ = significantly different from the early presence/later absence class. ^4^ = significantly different from the consistently present class. * *p* <.05, ** *p* <.01, *** *p* <.001

For boys, at age 9, there were no significant differences in parent-reported social comparisons across classes (all *p*s > 0.05, Cohen’s *d*s = 0.021–0.152). At age 15, there was one difference in child-reported social comparisons (see Fig. 5). Specifically, boys whose fathers were absent early but returned to the household later reported earlier timing (*M*_*timing*_ = 1.922, *SD* = 0.678) compared to same-aged, same-sexed peers than boys whose fathers were consistently present (*M*_*timing*_ = 2.139, *SD* = 0.678. class 2 vs. 4: *p* =.041, Cohen’s *d* = 0.218).

### Mediation analyses

Multiple mediation analyses were conducted to examine whether age 5 material hardship, father depression, and child BMI mediated associations between class membership and each indicator of pubertal timing (except pubic and underarm hair given the nonsignificant direct effects). The results of the multiple mediation analysis for thelarche and facial hair growth are depicted in Table 2. For girls, none of the proposed mediators significantly explained the association between father absence and thelarche and all indirect effects were nonsignificant (all *p*s > 0.17). However, several direct effects emerged, including class-specific associations between material hardship and thelarche at age 9 (*ps* ranging from 0.03 to 0.002). Similarly, for boys, none of the proposed mediators significantly explained the association between father absence and facial hair growth and all indirect effects were nonsignificant (all *p*s > 0.05). Again, several direct effects emerged. Across certain classes, higher child BMI and higher father depression were each associated with increased odds of facial hair growth at age 9 (*p*s ranging from 0.05 to 0.01).

These results were generally consistent with those found using social comparisons of puberty at age 9 and 15. None of the proposed mediators significantly mediated the association between father absence and social comparisons of puberty at either age for either sex (all *p*s > 0.71). Again, there were several direct effects. Among girls, class-specific associations between higher father depression and higher child BMI were associated with earlier pubertal timing (*p*s ranging from 0.05 to 0.001). Among boys, across certain classes, higher father depression, higher material hardship, and higher child BMI were each associated with earlier pubertal timing (*p*s ranging from 0.008 to 0.001). See Supplemental Table 3 for all direct effects.

**Table 2:** Results of multiple mediation analysis for girls (thelarche) and boys (facial hair)

Direct effect	β (SE)	t	*p*	LLCI, ULCI
Thelarche				
*Class 1: Consistently absent*				
Child BMI	0.349 (0.445)	0.785	0.433	−0.523, 1.221
** Material hardship**	**0.469 (0.147)**	**3.118**	**0.002**	**0.172**,** 0.748**
Depression	−0.025 (0.089)	−0.281	0.779	−0.200, 0.150
*Class 2: Early absence/Later presence*				
Child BMI	−0.195 (1.110)	−0.176	0.860	−2.372, 1.981
** Material hardship**	**−0.978 (0.467)**	**−2.094**	**0.036**	**−1.893**,** −0.063**
Depression	−0.298 (0.330)	−0.902	0.367	−0.944, 0.349
*Class 3: Early presence/Later absence*				
Child BMI	−0.539 (0.691)	−0.780	0.436	−1.894, 0.816
Material hardship	0.270 (0.254)	1.061	0.289	−0.228, 0.768
Depression	−0.024 (0.105)	−0.229	0.819	−0.230, 0.182
*Class 4: Consistently present*				
Child BMI	−0.481 (0.357)	−1.346	0.178	−1.181, 0.219
Material hardship	0.244 (0.195)	1.250	0.211	−0.138, 0.626
Depression	−0.067 (0.094)	−0.714	0.475	−0.252, 0.117
Facial hair growth				
*Class 1: Consistently absent*				
** Child BMI**	**−0.160 (0.081)**	**−1.963**	**0.050**	**−0.319**,** 0.000**
Material hardship	0.001 (0.002)	2.77	0.724	0.004, 0.022
** Depression**	**0.057 (0.022)**	**2.571**	**0.010**	**0.014**,** 0.101**
*Class 2: Early absence/Later presence*				
Child BMI	−0.110 (0.247)	−0.447	0.655	−0.594, 0.373
Material hardship	0.005 (0.005)	0.35	0.328	−0.021, 0.030
Depression	0.063 (0.080)	0.784	0.433	−0.094, 0.220
*Class 3: Early presence/Later absence*				
Child BMI	0.071 (0.108)	0.657	0.511	−0.141, 0.282
Material hardship	0.000 (0.008)	1.95	0.971	0.000, 0.031
Depression	0.065 (0.039)	1.667	0.095	−0.011, 0.142
*Class 4: Consistently present*				
** Child BMI**	**−0.203 (0.077)**	**−2.655**	**0.008**	**−0.353**,** −0.053**
** Material hardship**	**0.009 (0.008)**	**1.92**	**0.045**	**0.000**,** 0.018**
Depression	0.049 (0.026)	1.872	0.061	−0.002, 0.099

Significant effects at *p* <.05 were bolded for ease of interpretation. *β*: unstandardized beta value. *SE*: Standard error. *t*: t-value. *p*: significance level. *LLCI*: Lower limit confidence interval. *ULCI*: Upper limit confidence interval

## Discussion

Fathers matter. In the present study, we investigated how changes in father absence over infancy and childhood were associated with pubertal timing across sexes in a large, prospective sample of at-risk youth. We found that girls and boys whose fathers were consistently absent or who were absent early in life were more likely to report thelarche and facial hair growth at age 9 although results were not always consistent across different pubertal milestones. These findings suggest that father presence, particularly during the first years of life, may play an important role in shaping the timing of sex-specific pubertal indicators in both girls and boys. Although material hardship, father depression, and child BMI were examined as potential mediators, there was limited evidence for mediation, suggesting that these indicators may function as independent correlates, rather than explanatory mechanisms, of earlier pubertal timing.

Our results are consistent with prior work identifying father absence as one of the most robust psychosocial predictors of earlier pubertal timing in girls [9, 17, 55, 56]. Additionally, our findings extend this literature in two important ways. First, we demonstrate links between father absence and boy’s pubertal timing. Previous findings for boys have been inconsistent, sometimes finding that absence is linked with earlier pubertal timing and sometimes finding that it is linked with later timing [22]. These inconsistencies may reflect smaller sample sizes found in male studies of puberty or measurement limitations as boy’s pubertal development and early pubertal milestones are less salient and memorable than girl’s early pubertal milestones [12, 41]. Here, however, we used a large sample of boys with early measurement of socially salient milestones, finding that effects related to father absence were generally consistent across boys and girls. This suggests that boys’ pubertal development may be more sensitive to family structure than previously assumed and should be investigated further with better measures of early pubertal milestones in boys.

Second, we find that the timing of father absence mattered across sexes. Specifically, the most robust associations between father absence and earlier pubertal timing were observed for youth with consistently absent fathers as well as those with fathers who were absent during the first five years of life *only*. This was true even if fathers later returned to the household, emphasizing the importance of early absence rather than culminative exposure alone. The first year of life may represent a developmentally sensitive window for neuroendocrine development. This period is marked by two key processes: mini-puberty (i.e., a surge in sex steroid hormones that mimics adolescent pubertal onset); [57] and the organization of the HPA axis [30]. Together, these neuroendocrine changes set the foundation for pubertal development and stress regulation respectively, and their maturation is vulnerable to both organizing and disorganizing influences such as household chaos or dysfunction [36]. These mechanisms should be investigated in future research. Additionally, future research would benefit from better distinguishing father absence during the fetal period from the postnatal period which was not possible in the present study [58, 59].

Alternatively, differences in the timing of father absence may reflect patterns of non-resident father contact or broader family instability. Family dissolution is often accompanied by declines in father contact and involvement with their children [60]. When family dissolution occurs early in childhood or before birth, fathers may contribute fewer economic resources to the household, have less time to become involved in child rearing, or spend less time with their children [61, 62]. Because these associations were also observed among youth whose fathers later returned to the household, they could also reflect general family instability (e.g., cases where fathers are transitioning in and out of the household). In unstable family environments, caregiving burdens often fall solely on mothers, potentially reducing maternal sensitivity and increasing household stress and chaos [63]. Importantly, the current study cannot differentiate between father absence and father contact or parenting quality; it also cannot differentiate between stressors specific to father absence and those reflecting broader stress and adversity, all of which should be investigated in future research.

Notably, findings also differed across pubertal milestones and were most robust for thelarche in girls and facial hair growth in boys. Both milestones are linked to gonadarche; however, thelarche occurs early in pubertal development for girls whereas facial hair growth occurs later in development for boys [64]. Thus, pubic and underarm hair growth were also investigated as indicators that may be more comparable across sex, but they were not associated with patterns of father absence. Pubic and underarm hair growth primarily represent adrenarche, a separate physiological process that begins earlier in childhood, but whose physical manifestation occurs after the earlier signs of gonadarche [65]. To date, only three studies have investigated links between adrenarche and family structure; two reported no association and one reported an association for girls not boys [66–68]. Adrenal development is associated with later mood, internalizing symptoms, and even brain development;[69, 70] thus, future research would benefit from more precise, concurrent measurement of adrenal development to clarify whether and how different milestones relate to patterns of father absence.

Among the social comparison measures, we observed a pattern consistent with a neuroendocrine “recalibration” among girls but not boys [71]. Specifically, girls whose fathers were absent early but returned to the household later in childhood reported later relative pubertal timing at age 15 than those whose fathers were present early but absent later in childhood. Potentially, father’s return to the household and presence during middle childhood may recalibrate reproductive and stress-related systems, leading to more typical pubertal timing [71]. Indeed, some recent evidence suggests that early parent-child separation dysregulates HPA axis stress reactivity, but that subsequent parent-child reunions recalibrate these stress systems towards more typical reactivity [72]. This theory has not yet been extended to individual variation in pubertal timing, but it is a ripe space for future research. Despite its potential, we also urge caution in interpreting this result: It was not replicated across gender or other pubertal milestones; thus, it may be function of measurement choice or simply imply that social comparisons become less salient near the end of puberty.

We also conducted mediation analyses to examine whether material hardship, father mental health, and child BMI contributed to the observed relationships between father absence and pubertal timing. These variables were selected because they represent plausible pathways through which early family contexts could influence biological maturation—by shaping children’s stress exposure, nutritional environments, and psychosocial stability [17, 37, 68]. However, aligned with previous research,[25] we did not detect significant indirect effects for any of these putative mechanisms in either girls or boys. This could suggest that the link between father absence and pubertal timing may operate through other, unmeasured mechanisms. Alternatively, the timing of different effects as well as measurement error could have reduced our power to detect indirect effects in the current sample. Specifically, fathers reported on their own mental health and material hardship; although we supplemented the father report with mother reports when available, father absence may have introduced selection effects, systematically biasing the results. Future research should continue to investigate additional and potentially modifiable pathways—such as parenting quality, exposure to family conflict, or children’s physiological stress regulation—that may explain *how* and *why* early father absence influences the timing of pubertal development.

There are several limitations to consider. First, father absence was reported by mothers and did not account for time spent with the child or the quality of the father-child relationship. Although (when available) father reports of their own presence were consistent with mother reports, father presence alone does not fully capture a father’s impact on their children and non-residential fathers may still maintain a strong, positive presence in the child’s life [73]. Second, findings did not always replicate across pubertal milestones though they trended in similar directions. Divergence across pubertal milestones may reflect differences in measurement reliability and validity, or in perceptions of parents and children at different ages, highlighting the importance of considering multiple pubertal milestones assessed by different reporters.

Third, although our study includes a large sample of U.S. families, significant, and likely non-random, attrition occurred [74]. Additionally, missing data from fathers who were absent are unlikely to be missing at random, as absence is often associated with greater depression or material hardship. This pattern of missingness may lead to underestimation of the mediating role of these factors. Further, the cohort used is largely urban and socioeconomically disadvantaged so finding may not generalize to more advantaged, rural, or non-U.S. populations who have access to universal healthcare and robust social welfare systems. Future work would benefit from integrating or harmonizing multiple datasets to examine larger and more representative samples. Fourth, effects, when present, were small. Small effects may reflect conservative estimates due to measurement limitations, heterogeneity in developmental pathways, and the use of single indicators of pubertal timing. Although the magnitude of association between father absence and pubertal timing was modest, the presence of consistent effects across this sample and other large samples suggests potential developmental relevance that warrants continued investigation. Finally, as with all observational research, our findings may be subject to residual or unmeasured confounding. Despite adjusting for several key covariates, other factors such as genetic predispositions, prenatal exposures, or additional socioenvironmental influences, may also affect father absence and pubertal timing. This limitation is well documented in the literature and precludes causal inferences of these results [75].

Fathers play critical roles in their children’s health and development. The current study finds that girls and boys whose fathers were consistently absent or absent at birth and in the first year of life experienced earlier signs of pubertal development compared to girls and boys whose fathers were consistently present or present early but absent later. These findings emphasize the importance of early father presence, especially in the first year of life, which may be an important period for hormonal regulation and reproductive health. Notably, at later ages, the results also suggest that father reentry may be associated with later pubertal development, especially in girls, offering a potential area for further research.

## Supplementary Information

Supplementary Material 1

## Data Availability

The datasets generated and/or analyzed during the current study are available in the Future of Families and Child Wellbeing repository, https://ffcws.princeton.edu/.
