# Supplementary material for: Timing matters: a prospective investigation of father absence and pubertal timing in girls and boys
Source: Biol Sex Differ. 2026 Mar 19;17:89. doi: 10.1186/s13293-026-00868-y (PMC13122871; doi:10.1186/s13293-026-00868-y)
Supplement: Supplementary file 1 — Supplementary Material 1 [file 13293_2026_868_MOESM1_ESM.docx]

**Supplemental Material**

| **Table S1.** Fit Statistics for latent class models | | | | | | | | |
| --- | --- | --- | --- | --- | --- | --- | --- | --- |
| Model | Parameters | *LL* | *AIC* | *BIC* | a*BIC* | *Entropy* | *χ^2^ (p)* | *G² (p)* |
| 1-Class | 5 | -14319.08 | 28648.15 | 28680.63 | 28664.75 | 1.00 | 2889.77 (<0.001) | 304.01 (<0.001) |
| 2-Class | 11 | -10589.82 | 21201.64 | 21273.10 | 21238.15 | 0.85 | 847.92 (<.001) | 550.46 (0.03) |
| 3-Class | 17 | -5016.44 | 10066.87 | 10164.76 | 10110.75 | 0.79 | 255.44 (<.001) | 175.73 (<.001) |
| **4-Class** | **23** | **-4935.76** | **9917.53** | **10049.97** | **9976.89** | **0.77** | **12.61 (0.14)** | **14.38 (0.04)** |
| 5-Class | 29 | -4929.08 | 9916.16 | 10083.15 | 9991.01 | 0.78 | 0.84 (0.36) | 0.60 (0.25) |

Notes. *LL*: Loglikelihood; *AIC*: Akaike Information Criterion; *BIC*: Bayesian Information Criteria; a*BIC*: Adjusted BIC; *χ* ^2^: Pearson Chi-Square; *G²*: Likelihood Ratio Chi-Square. *p*: significance level. Bolded class represents the final solution.

**Table S2.** Father presence across time by class

|  |  | Birth | Year 1 | Year 3 | Year 5 | Year 9 |
| --- | --- | --- | --- | --- | --- | --- |
| Class | *n* | *%* | *%* | *%* | *%* | *%* |
| Consistently absent | 1812 | 6.2 | 1.6 | 1.5 | 2.0 | 16.0 |
| Early absence/Later presence | 398 | 20.5 | 9.8 | 53.6 | 95.6 | 92.3 |
| Early presence/Later absence | 846 | 67.7 | 65.6 | 40.5 | 21.4 | 44.6 |
| Consistently present | 1842 | 75.6 | 95.7 | 96.6 | 100.0 | 96.4 |

*Notes. n:* sample size. *%:* percentage

**Table S3.** Results of multiple mediation analysis for social comparisons of puberty

| Direct effect | *β* *(SE)* | *t* | *p* | *LLCI, ULCI* |
| --- | --- | --- | --- | --- |
| Girls’ social comparisons of puberty (age 9) | | | | |
| *Class 1: Consistently absent* |  |  |  |  |
| Child BMI | -0.183 (0.158) | -1.155 | 0.248 | -0.493, 0.127 |
| Material Hardship | -0.066 (0.038) | -1.726 | 0.084 | -0.140, 0.008 |
| **Depression** | **-0.158 (0.043)** | **-3.671** | **0.000** | **-0.242, -0.074** |
| *Class 2: Early absence/Later presence* |  |  |  |  |
| Child BMI | 0.024 (0.342) | 0.070 | 0.944 | -0.646, 0.694 |
| Material Hardship | 0.067 (0.088) | 0.769 | 0.442 | -0.105, 0.239 |
| **Depression** | **-0.250 (0.085)** | **-2.939** | **0.003** | **-0.417, -0.083** |
| *Class 3: Early presence/Later absence* |  |  |  |  |
| Child BMI | -0.234 (0.222) | -1.056 | 0.291 | -0.669, 0.201 |
| Material Hardship | -0.047 (0.060) | -0.783 | 0.434 | -0.165, 0.071 |
| Depression | -0.166 (0.086) | -1.929 | 0.054 | -0.335, 0.003 |
| *Class 4: Consistently present* |  |  |  |  |
| Child BMI | -0.067 (0.144) | -0.467 | 0.641 | -0.349, 0.215 |
| Material Hardship | 0.024 (0.042) | 0.562 | 0.574 | -0.058, 0.106 |
| **Depression** | **-0.170 (0.059)** | **-2.892** | **0.004** | **-0.286, -0.054** |
| Girls’ social comparisons of puberty (age 15) | | | | |
| *Class 1: Consistently absent* |  |  |  |  |
| Child BMI | 0.029 (0.143) | 0.203 | 0.839 | -0.251, 0.309 |
| Material Hardship | 0.035 (0.036) | 0.973 | 0.330 | -0.036, 0.106 |
| **Depression** | **-0.120 (0.043)** | **-2.798** | **0.005** | **-0.204, -0.036** |
| *Class 2: Early absence/Later presence* |  |  |  |  |
| **Child BMI** | **0.775, (0.380)** | **2.042** | **0.041** | **0.030, 1.520** |
| Material Hardship | 0.138 (0.092) | 1.488 | 0.137 | -0.042, 0.318 |
| Depression | -0.149 (0.107) | -1.390 | 0.165 | -0.359, 0.061 |
| *Class 3: Early presence/Later absence* |  |  |  |  |
| Child BMI | -0.035 (0.221) | -0.159 | 0.874 | -0.468, 0.398 |
| Material Hardship | 0.002 (0.064) | 0.039 | 0.969 | -0.123, 0.127 |
| Depression | -0.074 (0.088) | -0.849 | 0.396 | -0.246, 0.098 |
| *Class 4: Consistently present* |  |  |  |  |
| Child BMI | -0.174 (0.132) | -1.319 | 0.187 | -0.433, 0.085 |
| Material Hardship | 0.007 (0.038) | 0.175 | 0.861 | -0.067, 0.081 |
| Depression | -0.104 (0.054) | -1.932 | 0.053 | -0.210, 0.002 |
| Boys’ social comparisons for puberty at age 9 | | | | |
| *Class 1: Consistently absent* |  |  |  |  |
| Child BMI | 0.174 (0.175) | 0.995 | 0.320 | -0.169, 0.517 |
| Material Hardship | 0.036 (0.040) | 0.903 | 0.367 | -0.042, 0.114 |
| **Depression** | **-0.144 (0.046)** | **-3.121** | **0.002** | **-0.234, -0.054** |
| *Class 2: Early absence/Later presence* |  |  |  |  |
| Child BMI | 0.069 (0.536) | 0.129 | 0.897 | -0.982, 1.120 |
| Material Hardship | -0.141 (0.188) | -0.753 | 0.451 | -0.509, 0.227 |
| Depression | -0.251 (0.211) | -1.194 | 0.233 | -0.665, 0.163 |
| *Class 3: Early presence/Later absence* |  |  |  |  |
| Child BMI | -0.391 (0.273) | -1.434 | 0.151 | -0.926, 0.144 |
| Material Hardship | 0.025 (0.076) | 0.325 | 0.745 | -0.124, 0.174 |
| **Depression** | **-0.227 (0.086)** | **-2.638** | **0.008** | **-0.396, -0.058** |
| *Class 4: Consistently present* |  |  |  |  |
| Child BMI | -0.232 (0.163) | -1.426 | 0.154 | -0.551, 0.087 |
| Material Hardship | -0.023 (0.049) | -0.458 | 0.647 | -0.119, 0.073 |
| **Depression** | **-0.250 (0.060)** | **-4.157** | **0.001** | **-0.368, -0.132** |
| Boys’ social comparisons for puberty at age 15 | | | | |
| *Class 1: Consistently absent* |  |  |  |  |
| Child BMI | 0.090 (0.137) | 0.652 | 0.514 | -0.178, 0.358 |
| Material Hardship | -0.019 (0.034) | -0.553 | 0.581 | -0.086, 0.048 |
| Depression | -0.022 (0.041) | -0.551 | 0.581 | -0.102, 0.058 |
| *Class 2: Early absence/Later presence* |  |  |  |  |
| Child BMI | 0.629 (0.348) | 1.809 | 0.071 | -0.053, 1.311 |
| **Material Hardship** | **0.199 (0.094)** | **2.124** | **0.034** | **0.015, 0.383** |
| Depression | -0.118 (0.110) | -1.067 | 0.286 | -0.334, 0.098 |
| *Class 3: Early presence/Later absence* |  |  |  |  |
| Child BMI | -0.167 (0.193) | -0.864 | 0.387 | -0.545, 0.211 |
| Material Hardship | -0.039 (0.052) | -0.753 | 0.451 | -0.141, 0.063 |
| **Depression** | **-0.139 (0.069)** | **-2.020** | **0.043** | **-0.274, -0.004** |
| *Class 4: Consistently present* |  |  |  |  |
| **Child BMI** | **-0.297 (0.105)** | **-2.830** | **0.005** | **-0.503, -0.091** |
| Material Hardship | -0.006 (0.033) | -0.176 | 0.860 | -0.071, 0.059 |
| Depression | -0.011 (0.049) | -0.228 | 0.820 | -0.107, 0.085 |

*Notes.* Bolded rows are significant at *p* < .05 for ease of interpretation. All direct effects on the outcome (social comparisons of puberty at age 9 and 15) are depicted. There were no significant indirect effects. *β:*unstandardized beta value. *SE:* Standard error. *t:* t-value. *p*: significance level. *LLCI:* Lower limit confidence interval. *ULCI:* Upper limit confidence interval.

**Figure S1.** Father Presence and Girls’ Pubic and Underarm Hair Growth at Age 9. The relationship between father presence across five time points (birth, ages 1, 3, 5, and 9) and girls’ pubic and underarm hair growth at age 9 for each latent class. The solid blue line indicates the percent of fathers present across time points, the purple diamond marker represents the estimated percentage of individuals within each latent class who experienced pubic and underarm hair growth at age 9. A.) 72.7% of girls in the consistent absent class reported pubic and underarm hair at year 9. B.) 88.8% of girls in the early absence/later presence class reported pubic and underarm hair at year 9. C.) 74.6% of girls in the early presence/later absence class reported pubic and underarm hair at year 9. D.) 67.8% of girls in the consistently present class reported pubic and underarm hair at year 9. Subscripts represent significant differences between latent classes. ^1^ = significantly different from the consistently absent class. ^2^ = significantly different from the early absence/later presence class. ^3^ = significantly different from the early presence/later absence class. ^4^ = significantly different from the consistently present class. * *p* < .05, ** *p* < .01, *** *p* < .001

**Figure S2.** Father Presence and Boys’ Pubic and Underarm Hair Growth at Age 9. The relationship between father presence across five time points (birth, ages 1, 3, 5, and 9) and boys’ pubic and underarm hair at age 9 for each latent class. The solid blue line indicates the percent of fathers present across time points, the purple diamond marker represents the estimated percentage of individuals within each latent class who experienced pubic and underarm hair at age 9. A.) 17.7% of boys in the consistent absent class reported pubic and underarm hair at year 9. B.) 16.3% of boys in the early absence/later presence class reported pubic and underarm hair at year 9. C.) 12.5% of boys in the early presence/later absence class reported pubic and underarm hair at year 9. D.) 19.4% of boys in the consistently present class reported pubic and underarm hair at year 9. Subscripts represent significant differences between latent classes. ^1^ = significantly different from the consistently absent class. ^2^ = significantly different from the early absence/later presence class. ^3^ = significantly different from the early presence/later absence class. ^4^ = significantly different from the consistently present class. * *p* < .05, ** *p* < .01, *** *p* < .001
